# Malunion in a Self-stabilized Fracture of the Odontoid Process Type II with a Chronic Anterior Atlantoaxial Subluxation in a Neurologically Intact Patient: A Case Report

**DOI:** 10.1055/s-0043-1770979

**Published:** 2024-12-27

**Authors:** Juan Carlos Gomez-Rios, Claudio Uribe-Alpizar, Alejandro Reyes-Sanchez, Carla Lisette García-Ramos, Luis Miguel Rosales-Olivarez, Baron Zárate-Kalfópulos

**Affiliations:** 1 Serviço de Cirurgia de Coluna, Instituto Nacional de Rehabilitación, Cidade do México, México

**Keywords:** atlanto-axial joint, fractures, bone, odontoid process, spinal fractures

## Abstract

Atalanto-occipital dislocations with type II fractures of the odontoid process are rare, reporting 7 cases for every 784 upper cervical spine injuries, an incidence of <0.3% and are related to a high rate of morbidity and mortality. Regarding C2 fractures, the most common are in the odontoid process, representing 7%, classified by Anderson and D'Alonso according to their level, with the highest rate of pseudarthrosis in zone II of up to 85% are caused mainly by car accidents. In the acute event, there is no consensus regarding its optimal management. We performed a complete anamnesis and a physical examination in our institution. A systematic review of case reports was carried out with the keywords “mal-union, type II odontoid process fracture, chronic atlantoaxial subluxation” in four different databases, and a comparative analysis of the cases found was performed. There were not found identical cases to the one in our report, 9 similar case reports were published with the general differences of a pseudarthrosis of the odontoid process, as well as neurological alterations in symptomatic patients and the consequent surgical treatment.

Observing a patient with a type II odontoid process fracture consolidating viciously and a stable anterior atlantoaxial subluxation without neurological alterations is rare. We determined to maintain expectant management with annual follow-up.

## Introduction

Atalanto-occipital dislocations associated with type II fractures of the odontoid process are very rare injuries, reporting 7 cases for every 784 upper cervical spine injuries, an incidence <0.3%, 1 and are related to a high rate of morbidity and mortality due to its proximity to vital structures. Regarding C2 fractures, the most common are in the odontoid process, representing 7%. 2 Anderson and D'Alonso classified those fractures according to their level, with the highest rate of pseudarthrosis in zone II. Up to 85% 3 are caused by car accidents and there is no consensus regarding its optimal management, however, there are various surgical techniques for stabilization. If these fractures cannot be reduced by traction, they may require anterior decompression or transoral odontoid release followed by a posterior C1–C2 instrumented fusion. Several authors have reported on the utility of the retropharyngeal approach for the release of chronic angulated odontoid fractures and Rehman et al. 4 proved it to be an effective alternative in patients with old odontoid fractures, reporting an improvement of the postoperative JOA Score.

## Case Report

The Case Report was approved by the ethics committee of our institution under number INRLII/CI/141/21 on March 25, 2021.

A 21-year-old male patient, with no relevant history, began his condition on June 24, 2021, while driving a motorcycle on public roads, he skidded and collided with a retaining wall getting projected, without remembering the mechanism of injury, he was transferred by paramedics to a general hospital, where he received primary stabilization and the diagnosis of acute abdomen, type II odontoid process fracture and atlantoaxial subluxation. They performed a cholecystectomy and a left nephrectomy, and he stayed in the intensive care unit for 2 weeks where they proposed surgical treatment for the odontoid process fracture. However due to a lack of material, they opted for conservative management, and he was discharged on August 15, 2021, with the use of a rigid collar.

**Fig. 1:**
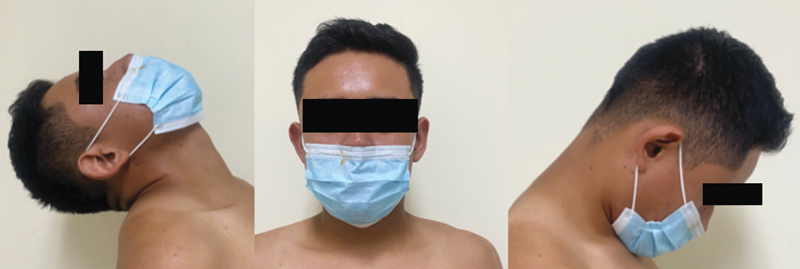
Arcs of mobility.

He was reassessed at the said institution on 08.29.21 where the follow-up of the conservative treatment was provided, and they send him to our institution to receive definitive surgical treatment due to saturation. Initially assessed in August 2022 with a clinical evaluation and, in the anamnesis he refers to being completely asymptomatic at that moment, working as a bricklayer despite being told to stop working, in the neurological examination without motor or sensory alterations, with adequate sphincter control, with arches complete active/passive mobility ( Fig. 1 ).

**Fig. 2:**
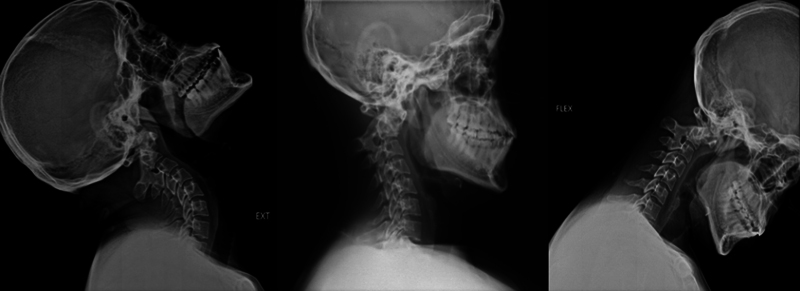
Dynamic radiographs of sagittal planes.

**Fig. 3:**
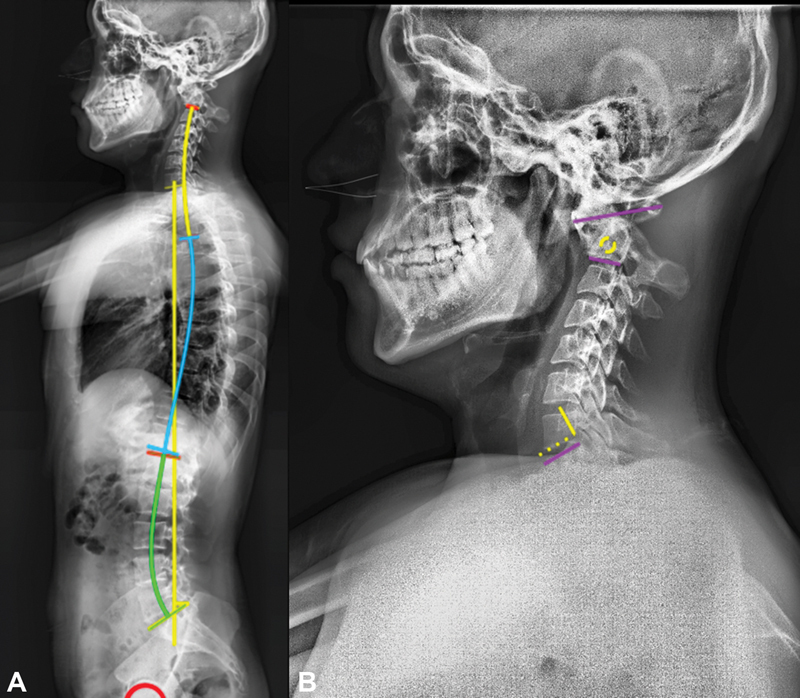
( **A** ) Spinopelvic parameters. ( **B** ) Cervical parameters.

**Fig. 4:**
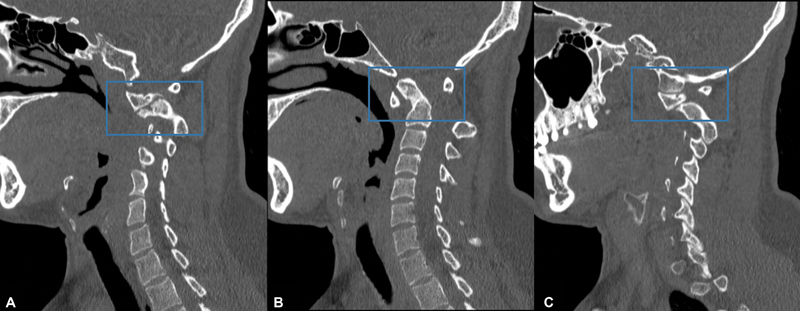
Tomography.

**Fig. 5:**
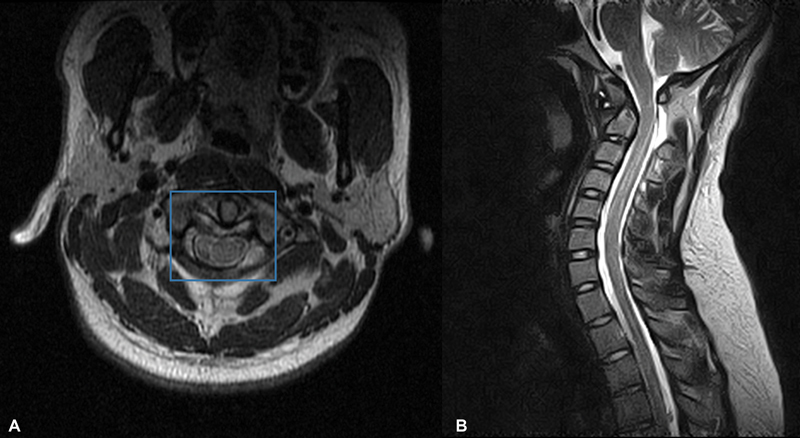
Magnetic resonance.

We performed an imaging assessment using X-rays, tomography, and magnetic resonance imaging with the following findings:

- Radiographs: The dynamic radiographic projections of the cervical spine ( Fig. 2 ) present a subluxation of C1 on C2 without changes in flexion or extension. Despite the injury, the panoramic radiographs ( Fig. 3A ) had normal sagittal balance and a normal pelvic-spinal balance for the Mexican population. 5 In the cervical sagittal axis ( Fig. 3B ) there is a subaxial hyperlordotic compensation that results from keeping the head upright and facing forward after the lesion, with the consequent overload of the facets at said levels.
- Tomography: In the sagittal cuts, a complete bone consolidation of the odontoid process is observed with sequelae of a fracture in zone II ( Fig. 4B ), it presents a subluxation of the right C1-C2 facet with the formation of a bone cay ( Fig. 4A ) and a subluxation of the left C1-C2 facet ( Fig. 4C ).
- Axial section Magnetic Resonance ( Fig. 5A ). The integrity of the transverse ligament is observed, with an adequate diameter of the medullary canal, in the sagittal cut ( Fig. 5B ) it is observed how the medulla freely runs through the medullary canal without compression.

## Discussion

Due to the rarity of this combination of lesions, there is no consensus regarding the optimal management of acute injury, much less the management of sequelae. A systemic review was carried out in the Cochrane, Clinicalkey, Ovid, and Pubmed databases with the words “malunion of odontoid process + chronic atlantoaxial subluxation” without finding any identical case, 14 “similar” case reports found in PubMed.

The main difference between the previous reports is that the patients treated were symptomatic or with some type of neurological alteration in addition to C2 pseudoarthrosis, reasons for which they decided to undertake surgical treatment. 6 7 8 9 10 On the other hand, there is a case report with lesser symptomatology treated with cervical traction, in whom two-week post-removal of traction developed gait disturbances, Grade 1 myelopathy, and worsening of the previous irreducible atlantoaxial dislocation showed by X-ray. 11 For those reasons, when we observed that the patient did not have neurological alterations, a consolidated fracture as well as stability of the subluxation, it was decided to refrain from performing any intervention and continue with the annual follow-up of the patient.

This combination of lesions is extremely rare, due to its high mortality, but a self-stabilization with consolidation and stability occurred, asymptomatically. As the only alteration, the patient presents a cervical hyperlordosis as part of the alteration of the cervical sagittal balance. Finally, due to the clinical characteristics, we decided on expectant management and to wait for the appearance of complications secondary to the biomechanical alteration and its opportune treatment due to his time of appearance.
