# Supplementary material for: Malunion in a Self-stabilized Fracture of the Odontoid Process Type II with a Chronic Anterior Atlantoaxial Subluxation in a Neurologically Intact Patient: A Case Report
Source: Rev Bras Ortop (Sao Paulo). 2024 Dec 27;59(Suppl 2):e133–7. doi: 10.1055/s-0043-1770979 (PMC11679622; doi:10.1055/s-0043-1770979)
Supplement: Related texts

# Research article: Má consolidação em fratura autoestabilizada de tipo II do processo odontoide com subluxação atlantoaxial anterior crônica em paciente neurologicamente intacto: Relato de caso

**Keywords:** articulação atlantoaxial, fraturas da coluna vertebral, fraturas ósseas, processo odontoide

## Abstract

As luxações atlanto-occipitais com fraturas de tipo II do processo odontoide são raras, com relato de sete casos a cada 784 lesões da coluna cervical superior. Sua incidência é inferior a 0,3% e estas lesões estão relacionadas a uma alta taxa de morbidade e mortalidade. As fraturas de C2 mais comuns são no processo odontoide, representando 7% dos casos. Essas fraturas são classificadas por Anderson e D'Alonso de acordo com seu nível e o maior índice de pseudoartrose foi observado na zona II. Até 85% dessas lesões são causadas por acidentes automobilísticos. Não há consenso quanto ao tratamento ideal dos casos agudos. Realizamos anamnese completa e exame físico em nossa instituição. Fizemos uma revisão sistemática de relatos de casos com as palavras-chave “ *malunion of odontoid process + chronic atlantoaxial subluxation* ” (má consolidação do processo odontoide + subluxação atlantoaxial crônica) em quatro bases de dados diferentes e uma análise comparativa dos casos encontrados. Não observamos casos idênticos ao do nosso relato. Nove relatos de casos semelhantes foram publicados; as principais diferenças foram a pseudoartrose do processo odontoide, as alterações neurológicas em pacientes sintomáticos e o consequente tratamento cirúrgico.

É raro observar um paciente com fratura do processo odontoide de tipo II, consolidação viciosa e subluxação atlantoaxial anterior estável sem alterações neurológicas. Decidimos pela conduta expectante com acompanhamento anual.

## Introdução

As luxações atlanto-occipitais acompanhadas por fraturas de tipo II do processo odontoide são muito raras, com relatos de sete casos a cada 784 lesões da coluna cervical superior. Sua incidência é inferior a 0,3%; 1 estas lesões estão relacionadas a uma alta taxa de morbidade e mortalidade. As fraturas de C2 mais comuns são do processo odontoide, representando 7%. 2 Anderson e D'Alonso classificaram estas fraturas de acordo com seu nível e a maior taxa de pseudoartrose foi observado na zona II. Até 85% 3 destas lesões são causadas por acidentes automobilísticos e não há consenso quanto ao seu tratamento ideal, embora existam várias técnicas cirúrgicas para estabilização. Se essas fraturas não puderem ser reduzidas por tração, podem exigir descompressão anterior ou liberação transoral do odontoide seguida por fusão posterior instrumentada de C1-C2. Vários autores relataram a utilidade da abordagem retrofaríngea para a liberação de fraturas crônicas do odontoide angulado; Rehman et al. 4 comprovaram ser uma alternativa eficaz em pacientes com fraturas antigas do odontoide, relatando melhora da pontuação da *Japanese Orthopaedic Association* (JOA) pós-operatória.

## Relato de Caso

O relato de caso foi aprovado pelo comitê de ética de nossa instituição sob número INRLII/CI/141/21 em 25 de março de 2021.

Paciente do sexo masculino, 21 anos de idade, sem antecedentes relevantes, sofreu a lesão no dia 24 de junho de 2021; ao conduzir motocicleta em via pública, derrapou e colidiu com um muro de contenção ao se projetar. Sem lembrar o mecanismo da lesão, foi transferido por paramédicos para um hospital geral, onde foi submetido à estabilização primária e recebeu o diagnóstico de abdome agudo, fratura do processo odontoide de tipo II e subluxação atlantoaxial. Também foi submetido a uma colecistectomia e uma nefrectomia esquerda. O paciente permaneceu na unidade de terapia intensiva por 2 semanas, onde foi proposto o tratamento cirúrgico para a fratura do processo odontoide. Porém, por falta de material, optou-se por conduta conservadora e o paciente teve alta em 15 de agosto de 2021, com uso de colar rígido.

O paciente foi reavaliado na mesma instituição em 29 de agosto de 2021, onde ocorreu o acompanhamento do tratamento conservador, e foi encaminhado para nossa instituição para tratamento cirúrgico definitivo por saturação. Sua primeira avaliação clínica aconteceu em agosto de 2022 e, à anamnese, o paciente referia estar totalmente assintomático naquele momento, trabalhando como pedreiro, apesar de ter sido orientado a parar de trabalhar. Ao exame neurológico, o paciente não apresentava alterações motoras ou sensoriais e tinha controle esfincteriano adequado e amplitude de movimento ativa/passiva completa ( Fig. 1 ).

**Fig. 1:** Amplitude de movimento.

As técnicas de diagnóstico por imagem (radiografia, tomografia computadorizada e ressonância magnética) revelaram os seguintes achados:

- Radiografias: As projeções radiográficas dinâmicas da coluna cervical ( Fig. 2 ) mostraram uma subluxação de C1 em C2 sem alterações em flexão ou extensão. Apesar da lesão, as radiografias panorâmicas ( Fig. 3A ) revelaram equilíbrio sagital e espinopélvico normal para a população mexicana. 5 No eixo sagital cervical ( Fig. 3B ), havia uma compensação hiperlordótica subaxial decorrente da manutenção da cabeça ereta e voltada para frente após a lesão, com consequente sobrecarga das facetas nesses níveis.
- Tomografia: Nos cortes sagitais, observou-se consolidação óssea completa do processo odontoide com sequela de fratura na zona II ( Fig. 4B ), além subluxação da faceta C1-C2 direita com formação de lesão óssea ( Fig. 4A ) e subluxação da faceta esquerda de C1-C2 ( Fig. 4C ).
- Ressonância magnética em corte axial ( Fig. 5A ): Havia integridade do ligamento transverso e diâmetro adequado do canal medular. Em corte sagital ( Fig. 5B ), observou-se que a medula corria livremente pelo canal medular, sem compressão.

**Fig. 2:** Radiografias dinâmicas em planos sagitais.

**Fig. 3:** ( **A** ) Parâmetros espinopélvicos. ( **B** ) Parâmetros cervicais.

**Fig. 4:** Tomografia computadorizada.

**Fig. 5:** Ressonância magnética.

## Discussão

Devido à raridade dessa combinação de lesões, não há consenso sobre seu tratamento ideal em casos agudos, muito menos sobre o manejo das sequelas. Uma revisão sistemática foi realizada nas bases de dados Cochrane, ClinicalKey, Ovid e Pubmed com as palavras-chave “ *malunion of odontoid process + chronic atlantoaxial subluxation* ” (má consolidação do processo odontoide + subluxação atlantoaxial crônica); não encontramos nenhum caso idêntico, mas 14 relatos de casos “semelhantes” no PubMed.

A principal diferença quanto aos relatos anteriores é que os pacientes tratados eram sintomáticos ou apresentavam algum tipo de alteração neurológica além da pseudoartrose de C2, motivos pelos quais optaram pelo tratamento cirúrgico. 6 7 8 9 10 Por outro lado, há um relato de caso com sintomatologia menor tratado com tração cervical. No entanto, 2 semanas após a retirada da tração, o paciente desenvolveu distúrbios de marcha, mielopatia de grau 1; além disso, uma radiografia demonstrou a piora da luxação atlantoaxial prévia, não passível de redução. 11 Por isso, quando observamos que o paciente não tinha alterações neurológicas e apresentava fratura consolidada e estabilidade da subluxação, optamos por não realizar qualquer intervenção e continuar com seu acompanhamento anual.

Esta combinação de lesões é extremamente rara devido à sua elevada mortalidade; porém, houve uma autoestabilização com consolidação e estabilidade de forma assintomática. Como única alteração, o paciente tinha hiperlordose cervical como parte da alteração do equilíbrio sagital cervical. Por fim, devido às características clínicas, optamos pela conduta expectante e por aguardar o aparecimento de complicações secundárias à alteração biomecânica, que serão tratadas de forma oportuna caso ocorram.
